# Fee Bill

**Published:** 1863-02

**Authors:** 


### FEE BILL.

The dentists of Cincinnati and Covington have framed and adopted a fee bill for professional services, to take effect from February 1st; it will be found on another page of this number. This we consider an important movement in several respects; in the first place it is an act of justice to the dentists themselves, in a pecuniary point of view, and by it they will be made better dentists. It is a movement of immense advantage to the people, for we feel assured, that their work will be much better done now than before, and they will prize it far more highly, than if it cost them but little, and was poorly done. The minimum fees of this bill are really no advance, upon what many of the dentists in the city have been charging heretofore; $3 00 is not as good now as $2 00 before the advance in gold. We are well assured, that some of our operators will not keep down to this level, but will make their average fees considerably above it. When gold comes up to the vicinity of one hundred per cent., no thorough operator can afford to fill teeth for less than $5 00

The query has once or twice been made, will the dentists all adhere to the fee bill? We know they will, for they can not do otherwise without robbing themselves. The movement should be a general one all over the country, else the dentists must suffer. T.

